# Trauma in the courtroom: The role of prior trauma exposure and mental health on stress and emotional responses in jurors

**DOI:** 10.1111/bjc.12522

**Published:** 2024-12-23

**Authors:** Matthew Brooks, Jessica Glynn, Hannah Fawcett, Aminah Barnes, Rachael Carew, David Errickson, Maria Livanou

**Affiliations:** ^1^ School of Psychology, Manchester Metropolitan University Manchester UK; ^2^ School of Life Sciences, Coventry University Coventry UK; ^3^ Cranfield Forensic Institute, Cranfield University Cranfield UK; ^4^ School of Psychology, University of Bolton Bolton UK

**Keywords:** jurors, jury duty, mental health, stress, trauma

## Abstract

**Objectives:**

Prior research indicates that jury duty can be distressing for some jurors. This study examined: (1) the influence of prior trauma characteristics (type, exposure, time since trauma), medical fear and mental health difficulties on stress and emotional responses during a mock trial and 1 week later; and (2) associations between early stress reactions during a trial on subsequent stress and emotional reactivity after exposure to skeletal evidence and 1 week later.

**Methods:**

Mock jurors (*n* = 180) completed baseline self‐report mental health measures, read a summary of a murder case and were then exposed to graphic skeletal evidence. Stress and/or emotional responses were collected at baseline, after reading the case summary, before and after viewing the skeletal evidence and 7 days post‐trial.

**Results:**

Participants reported a wide range of prior traumatic experiences, with nearly half reporting pre‐existing mental health difficulties. Average traumatic stress symptoms tripled from baseline to follow‐up, with 44% of participants meeting PTSD‐type criteria 7 days later. Medical fear and mental health difficulties were positively associated with some stress and/or emotional responses throughout the trial, with mixed findings concerning trauma characteristics, stress and emotional reactivity. Initial stress and emotional responses to case evidence were linked to later stress and emotional reactions, after accounting for pre‐existing trauma and mental health characteristics.

**Conclusions:**

Past trauma experiences, mental health difficulties and immediate stress responses during a trial can exacerbate emotional and stress reactions. Addressing the psychological impacts of pre‐existing trauma symptoms could improve juror well‐being during this important civic duty.

**Practitioner Points**

- The significant increase in PTSD symptoms among jurors over the course of the study highlights the need to facilitate access to immediate post‐trial support in England and Wales for those experiencing psychological distress.
- Jurors—especially those with prior trauma exposure—may experience emotional distress during court cases, and could benefit from support to help them psychologically prepare for trials.
- Identifying jurors at higher risk of stress symptoms due to self‐reported mental health and trauma experiences could inform the provision of specialist post‐trial support.
- Training professionals working in court settings on mental health and trauma is essential to recognize distress in jurors and themselves.

## INTRODUCTION

Jury duty in England and Wales is a civic responsibility that is an integral aspect of the criminal justice system. The Crown Court outstanding caseload stood at 67,600 at the end of December 2023 (Sturge, 2024), with the latest data on juror summons showing 488,000 jurors were summoned, with 136,000 sitting on a jury in 2023 (His Majesty's Courts and Tribunals Service, personal communication, October 24, 2024). [Correction added on 06 May, 2025, after First Online publication: The value ‘136,00’ is revised to ‘136,000’ in the previous sentence in this version.] These jurors are drawn from the electoral register and tasked with the solemn duty of participating in legal proceedings, weighing up evidence and testimonies and determining the guilt or innocence of the accused. Although participation in jury duty is an important and rewarding experience for some jurors (Gastil & Weiser, 2006; Thomas, 2020), this weight of responsibility, paired with the often harrowing and traumatic material jurors encounter during their service, can lead to harmful psychological effects for others (Welsh et al., 2020). Jurors may present with histories of personal trauma (Robertson et al., 2009), and stress responses may be further exacerbated because of their participation in trials, although this has received limited attention in the literature. The current study will investigate the influence of prior trauma exposure on stress and emotional responses in jurors, with a view to enhance support provided to individuals who fulfil this important duty.

### Context of jury duty in England and Wales

The current socio‐legal context of jury duty in England and Wales can present challenges for jurors, who have little say about exposure to traumatic disclosures, information or material through sitting on criminal cases. Before any trial has taken place, members of the public are legally compelled to serve on trials should they be summoned, and can only be excused in limited exceptional circumstances (GOV.UK, n.d.). Exemptions are in place for individuals that are resident in a hospital on account of mental disorder (as defined by the Mental Health Act, 1983), or who do not have mental capacity to make a decision (in line with the Mental Capacity Act, 2005) at the time of summons are ineligible for jury duty (Criminal Justice Act, 2003). Concerns have been raised (Ellison & Munro, 2017) that jurors receive limited psychological support throughout a trial, and minimal preparation in advance of undertaking jury duty. During the trial, jurors may be exposed to potentially distressing evidence in the form of testimony from victims and witnesses, and the presentation of forensic material (Grady et al., 2018); jurors are also prevented from discussing the case with anyone other than the jurors in the deliberation room (Juries Act 1974, S20D). When deciding on the guilt of the accused, jurors are asked to separate their own personal feelings towards the case before reaching a verdict. Following a trial, jurors are legally prevented from discussing their deliberations, although they may share aspects of their experience in the courtroom itself (Juries Act, 1974). Guidance signposts jurors requiring emotional support in the direction of their GP/family medical practitioner or the national Samaritans charity, although any help that could be provided is limited due to legal restrictions, perceptions of a lack of tailored support or jurors' uncertainty about what legally can and cannot be discussed (Thomas, 2020).

### Psychological consequences of jury duty

The psychological consequences of participating in jury duty has received sparse attention in the literature, and in wider public discourse (Thomas, 2020). Although some studies find favourable opinions of participating in jury duty (Bornstein et al., 2005; Thomas, 2020), other research has identified significant negative psychological aftereffects. Available evidence, largely from the United States, suggests that as many as 50% of jurors display trauma‐related symptoms, such as intrusive thoughts, anhedonia, loss of appetite and sleeping difficulties (Lonergan et al., 2016). These symptoms were found to persist in a minority of jurors for months after a trial ended. Within the United Kingdom, juror well‐being research is more limited. In a study of 64 self‐selecting jurors (Robertson et al., 2009), various aspects of jury duty, including feeling isolated from loved ones during the trial and being presented with disturbing evidence, were highlighted as sources of distress. These findings have since been replicated in other research conducted in Scotland (Welsh et al., 2020) which has a different legal system to that of England and Wales. In this study, it was reported that serving on longer trials, sitting on crimes against the person cases and being female, are all associated with greater distress. The largest study of UK jurors (*N* = 1175; Thomas, 2020) suggested that the majority found the experience to be positive (78% ‘interesting’, 58% ‘educational’, 55% ‘informative’), although a substantial minority (42%) viewed the experience as ‘stressful’. These findings, and high‐profile criminal cases in the media, have raised concerns among professional bodies (British Psychological Society [BPS], 2023) and policy makers (Hansard, 2023, 2024) of the need for better well‐being support for jurors.

### Prior trauma exposure, medical fear and mental health in jurors

Jurors are not only exposed to potentially traumatic events in the courtroom, but may themselves present with histories of personal trauma and mental health difficulties that could influence stress and emotional responses both before, during and after a trial. Research that has investigated prior traumatic experiences as a risk factor for stress symptoms in jurors is limited and has observed mixed findings. One study (Robertson et al., 2009) found that female jurors who sat on trials that were relevant to previous traumatic experiences reported significantly higher stress symptoms than male jurors with or without trauma. This finding could be contextualized within a vulnerability‐stressor perspective, in which exposure to stressful stimuli may elicit and exacerbate distress (Ingram & Luxton, 2005). Research has found that exposure to previous traumatic events and pre‐existing trauma symptoms can evoke stronger immediate symptomatic reactions to newly experienced traumatic events (Gould et al., 2021), suggesting that prior trauma exposure may place individuals at vulnerability for increased distress following further exposure to stressors. However, other research has identified similar stress symptoms in jurors with and without prior trauma exposure (Palmer, 2005). This finding could be due to the inoculating effects of prior exposure, which buffer individuals against potential negative symptoms from subsequent trauma (Seery, 2011). Alternatively, the context‐dependent nature of stress reactions, which may vary according to the type, frequency, severity and timing of trauma experienced (Gerber et al., 2018), and individual differences in coping responses (Breslau et al., 2008), may explain differences in reported stress among jurors with and without prior trauma experiences. Vulnerability‐stressor perspectives recognize individual predisposing factors (e.g. prior trauma exposure) can interact with the social and relational context. Within a courtroom, juror distress may be exacerbated by the pressure of reaching a verdict, the deliberation process with other jurors (Robertson et al., 2009) and the unpredictability and lack of control over courtroom processes (Carline et al., 2024).

The extent to which stress and emotional responses are influenced by trauma type has been understudied in the wider literature, with mixed findings. Some traumas can be categorized as interpersonal traumas if they are deliberately perpetrated acts committed by one person/group of people towards others, or there is an intention to cause harm, such as through criminal victimization (Mauritz et al., 2013). In contrast, non‐interpersonal traumas (e.g. natural disasters) are those outside of the control of individuals and occur without premeditation. Studies have shown that emotion and stress responses are heightened (Amstadter & Vernon, 2008; Kongshøj & Bohn, 2023) and more dysregulated (Berfield et al., 2022) in people with a history of interpersonal trauma, such as sexual victimization. Yet, other arguments suggest the type of trauma is less relevant compared to subjective reactions to the event in determining psychological outcomes (Boals, 2018). The extent to which jurors' stress and emotional responses are influenced by the type of trauma experienced is unknown and warrants further investigation to establish if further support is required for jurors with specific trauma profiles.

In addition to trauma type, the level of exposure to a traumatic event and the time since the event was experienced may influence stress and emotional responses. Although indirect exposure to traumatic events, such as through witnessing an event or hearing distressing disclosures from others, can lead to stress responses, such responses are heightened following direct exposure to trauma (May & Wisco, 2016). The time that has passed since a traumatic event may also influence stress and emotional responses. Trauma symptoms that persist longer than 1 month may indicate a diagnosis of post‐traumatic stress disorder (PTSD; American Psychiatric Association, 2013), although available evidence on the relationship between time since the index event and subsequent responses is mixed. The influence of trauma on stress and emotional responses may decrease with time but can depend on the type of trauma experienced (Izutsu et al., 2004) or vary as a function of the developmental periods in which traumas occurred (Dunn et al., 2018). However, one review found that time since trauma did not relate to psychological outcomes in adults exposed to adversity (Szabo et al., 2017). Given that negative trauma‐related beliefs can persist for long periods after the initial event (Cole et al., 2024) and for some people could reach diagnostic thresholds, more research is needed to ascertain whether juror stress and emotional responses may be influenced by the level of prior trauma exposure and the time since the event to help inform support efforts.

A history of mental health difficulties may place some jurors at greater risk of elevated stress/emotional responses. Under the Criminal Justice Act (2003), potential jurors may be excused due to ‘serious’ mental health difficulties, although this provision may disregard the proportion of subclinical mental health difficulties among the wider population (Baker & Kirk‐Wade, 2024). Literature on jurors has previously discussed mental health difficulties as a consequence rather than as a precursor to stress (Miller et al., 2007). However, it is possible that prior mental health difficulties could also be viewed as a risk factor for exacerbated stress and emotional responses to subsequent stressful situations, as mental health difficulties may be associated with challenges in regulating such responses (Berking & Wupperman, 2012). Similarly, anxiety towards medical procedures and paraphernalia such as needles and blood, referred to as medical fear (Olatunji et al., 2012), may have relevance within some criminal trials as jurors are exposed to potentially graphic materials that feature blood, wounds and skeletal remains. Medical fear has been associated with heightened emotional reactivity (Olatunji et al., 2012). Research that examines the role of prior mental health difficulties and medical fear on emotion and stress responses among jurors could highlight individuals who may require additional support before a trial involving graphic evidence commences.

### Rationale and aims of study

In recent years there have been calls (e.g. Ellison & Munro, 2017; Molnar et al., 2017) to further examine the psychological well‐being of populations exposed to potentially distressing situations. Jurors represent a population who are regularly exposed to harrowing material within criminal trials, and who may themselves present with trauma history and other mental health challenges prior to entering the courtroom. Available research has suggested that some jurors can be negatively affected by their participation in jury duty, although the impact of prior trauma exposure on juror well‐being has received even less attention. Research that has acknowledged prior trauma exposure in jurors has considered its influence on verdict decisions (Bottoms et al., 2017; Lilley et al., 2023), rather than stress or emotional responses. In line with the vulnerability‐stressor framework (Gould et al., 2021; Ingram & Luxton, 2005), it is possible that jurors with exposure to prior traumatic events and/or who have pre‐existing stress symptoms could be at increased risk for psychopathology following exposure to subsequent distressing material during a trial, and that exposure to such material may exacerbate stress symptoms in relation to index trauma previously experienced. Identifying the factors that contribute towards juror well‐being could help inform practice guidelines for more trauma‐informed courtroom practices, and raise awareness for tailored well‐being support for jurors, particularly those who may be vulnerable to more severe negative reactions.

The aims of this study were to investigate the extent to which prior traumatic experience characteristics (type of trauma [interpersonal vs. non‐interpersonal], trauma exposure [direct vs. indirect], time since the most severe trauma), medical fear and mental health difficulties are associated with: (1) pre‐trial mental health; (2) emotional and stress responses to case and skeletal evidence; (3) stress responses 1 week later among mock jurors. Furthermore, while statistically controlling for the effects of prior trauma and mental health characteristics, we examined whether early stress responses were associated with subsequent stress and emotional responses to further trauma exposures during the trial. Due to limited evidence, it was anticipated that trauma characteristics, medical fear and mental health difficulties would be associated with mock jurors' stress and emotional responses during and post‐trial. It was also hypothesised that early stress and emotional responses may exacerbate traumatic stress symptoms in relation to index trauma, and subsequent stress and emotional reactions following exposure to additional distressing material.

## METHOD

### Participants

A convenience sample of 180 jury‐eligible participants was recruited. Power analysis for multiple regression with nine predictors, 80% power, alpha of .05 and medium effect size (*f* = 0.15), suggested a minimum sample size of 114. Inclusion criteria were that participants must be eligible for jury duty in England and Wales (including being aged between 18 and 76 years old, registered to vote and have lived in the UK for at least 5 years after their 13th birthday, Juries Act, 1974). The sample was mostly female (67.2%), aged between 18 and 74 years (*M*_age_ = 38.33 years, SD = 15.61), had an undergraduate level of education (38.9%) and was in full‐time employment (47.2%). Around a fifth of participants (17.8%) had previously completed jury duty.

### Materials

#### Demographics

Information about the participants' demographic characteristics (including age, gender identity and details of their education/employment history) was collected through a self‐completed form, designed for the purposes of the present study.

#### Stress symptoms

##### Trauma exposure and post‐traumatic stress symptoms

PTSD symptoms over the past month were assessed using the PTSD Checklist for DSM‐5 (PCL‐5; Blevins et al., 2015), and over the past 7 days using the Impact of Events Scale‐Revised (IES‐R; Weiss & Marmar, 1997). The PCL‐5 is a 20‐item measure of DSM‐5 criteria PTSD symptoms experienced in the past month, on a scale from 0 (not at all) to 4 (extremely). An example item is ‘Repeated, disturbing, and unwanted memories of the stressful experience’. The overall PTSD symptom score for the measure was used in this study, with higher scores indicative of more severe PTSD symptoms. The PCL‐5 has demonstrated excellent psychometric properties (Blevins et al., 2015), and in this study, the internal consistency was high (*α* = .94). The PCL‐5 with criterion A was used, which also asked participants to indicate the most severe event they had experienced, how long ago it had occurred, whether it was experienced directly or indirectly and whether the event was an accident, violent or natural occurrence.

The IES‐R is a 22‐item measure of PTSD symptoms (e.g. ‘I had trouble staying asleep’) rated on a scale ranging from 0 (not at all) to 4 (extremely) across three subscales of intrusions, avoidance and hyperarousal. Elevated scores on these subscales indicate more severe PTSD symptoms. The measure has sound psychometric properties (Weiss & Marmar, 1997) and demonstrated high reliability in this study at baseline (*α* = .93) and day seven (*α* = .95). Participants were asked to respond to the questionnaire with reference to the most serious trauma they previously reported in the PCL‐5.

##### Perceived stress

The Perceived Stress Scale‐10 (PSS‐10; Cohen et al., 1983) is a 10‐item measure of the degree to which situations (e.g. ‘upset because of something that happened unexpectedly’) in a person's life are perceived as stressful. The referent period is the past month. Items are rated on a scale from 0 (never) to 4 (very often), with higher scores indicating greater perceived stress. The PSS‐10 has demonstrated good psychometric properties (Cohen & Williamson, 1988), showing acceptable reliability in this study at baseline (*α* = .88) and day seven (*α* = .84).

##### Subjective stress

**FIGURE 1:**
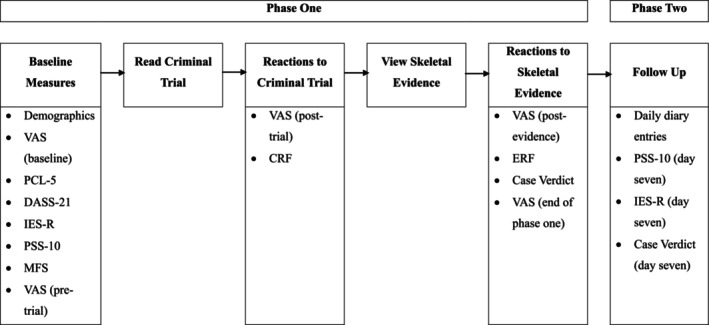
Outline of study procedure.

A visual analogue scale (VAS) was used to measure subjective stress at different points during the study (see Figure 1), on a scale ranging from 0 (not at all stressed) to 100 (very stressed).

#### Mental health difficulties

##### General mental health difficulties

The Depression, Anxiety and Stress Scales (DASS‐21; Lovibond & Lovibond, 1995) is a 21‐item measure of common mental health difficulties. Across three subscales (depression, anxiety, stress), the DASS‐21 evaluates participants' experiences of symptoms in these areas over the preceding week, using a rating scale ranging from 0 (did not apply to me at all) to 3 (applied to me very much or most of the time). Elevated scores indicate a greater presence of mental health difficulties. The DASS‐21 has shown satisfactory psychometric qualities (Lovibond & Lovibond, 1995) and demonstrated high reliability in this study (*α* = .94). Alongside this measure, participants were also asked to self‐report whether they had a history of mental health difficulties (yes/no).

##### Medical fear

The Medical Fear Survey (MFS; Olatunji et al., 2012) is a 25‐item measure of general fear and anxiety associated with medical information or conditions. Participants rate, on a scale from 0 (no fear or concerns) to 3 (intense fear), the extent to which they would be distressed by various medical situations, such as having blood drawn from an arm, or experiencing feelings of nausea. A total score was created, with higher scores indicating greater medical fear. The MFS has shown excellent convergent and discriminant validity (Olatunji et al., 2012), and displayed high internal reliability in this study (*α* = .90).

#### Emotional reactions

##### Emotional responses to the case

The Case Reactions Form (CRF; Livanou et al., 2024a) is a measure of emotional reactions created for the purposes of this study that was administered after reading the case evidence. The CRF was designed to illicit participant's emotional responses in relation to the materials presented, with the inclusion of responses such as helplessness and disgust not included in existing measures (e.g. Positive and Negative Affect Scales, Watson et al., 1988; State Trait Anxiety Inventory, Spielberger et al., 1983). On a scale from 0 (not at all) to 3 (extremely), participants were asked to rate the severity of 11 emotional reactions they had experienced (e.g. upset, angry, confused), with a total emotional reaction score created. Higher scores reflect more intense emotional responses. The ERF (*α* = .89) demonstrated acceptable reliability in the study. Separate from the 11 emotional responses, participants were asked about their ability to concentrate on the case with a single item on a scale from 0 (completely unable to concentrate) to 3 (able to concentrate very well). Participants were also asked about the extent to which they could vividly imagine the circumstances in the case, with a single item on a scale from 0 (not vividly at all) to 3 (extremely vividly).

##### Emotional responses to the skeletal evidence

The Evidence Reactions Form (ERF; Livanou et al., 2024b) consists of the same items and scoring as the CRF, but with amended instructions that asked participants to respond in relation to viewing the skeletal evidence. The ERF (*α* = .90) demonstrated high reliability.

### Procedure

The study received institutional ethics approval. Participants were recruited via local advertisements in universities, coffee shops, libraries and community centres and via dedicated social media channels on Facebook and LinkedIn. The recruitment materials made clear that participants would be required to act as a juror reading a murder case, examining associated human skeletal remains and completing a range of psychological measures. Potential participants were also encouraged not to participate if they found the nature of the study upsetting. Interested participants contacted the research team who provided an information sheet with details of the study and a consent form for participants to consider before agreeing to participate, This detailed their confidentiality and withdrawal rights as participants, and the voluntary nature of their involvement. Once participants provided informed consent, mutually agreeable dates were arranged for participation. All participation dates allowed for a two‐week cooling‐off period from the initial expression of interest to allow participants to fully consider the study requirements before deciding to take part. Participants were offered a choice of two venues for data collection to take place, either on the university campus or at a community venue local to the participant. At the venue, participants provided their consent again and data was collected using online questionnaires in the software Qualtrics, and can be found on the Open Science Framework: https://osf.io/x4k8m/.

The study consisted of two phases, summarized in Figure 1. Phase one refers to the in‐person exposure to case and evidential material and completion of study measures. Participants completed the measures, were exposed to evidence, and reached a verdict individually. Phase two refers to the diary element of the study whereby participants provided follow‐up data over 7 days. As with phase one, participants completed measures and reached a verdict on their own.

In phase one, which lasted approximately 45 min, participants completed a written consent form, provided demographic information and completed baseline psychological measures relating to trauma exposure (PCL‐5), stress (VAS, PSS‐10), PTSD (IES‐R), medical fear (MFS) and mental health symptoms (DASS‐21) using online survey software Qualtrics. Next, participants were given a mock murder case that was devised for the study, based upon a real‐life case. The case summary included instructions for the ‘jury’ and details of the crime, in which a 27‐year‐old female victim was found dismembered in a local park. Details were also provided in relation to a suspected offender, a 32‐year‐old male who pled not guilty. After reading the case, participants completed the VAS and CRF. Participants were then presented with skeletal evidence which featured signs of traumatic injury to a human skull. Participants were pseudo‐randomly allocated into one of the three condition groups using Microsoft Excel, ensuring equal numbers of participants in each of the three evidence presentation formats: (1) digital photographs of actual skeletal remains from an autopsy; (2) a moving virtual 3D reconstruction of a skull to watch on a computer; or (3) in the form of a 3D printed model, which could be held. The evidence was sourced and used with appropriate permissions from the Coroner's Office and the Deputy Director of Intelligence. Participants were able to freely examine the evidence for as long as they wished. After the evidence was presented, participants completed the VAS and ERF and provided a verdict on the case. To assess attention to the stimulus, there were regular breaks between completing questionnaires and assessing evidence (see Figure 1), reverse‐scored items on the study measures and participants were asked which evidence they had viewed and responded to some questions about the case (e.g. the name of the victim).

In phase two, following the day of initial data collection, participants received an email inviting them to complete a daily diary over a period of 7 days to explore the presence of more sustained psychological symptoms post‐trial. The daily diary required participants to complete the PSS‐10 and IES‐R, along with some open‐ended qualitative questions. Prior work (e.g. Arnaudova & Hagenaars, 2017) that has included a 7‐day follow‐up was able to identify trauma‐related symptoms during this relatively short period. Participants were also asked to provide a verdict at day seven, and compensated with a £10 shopping voucher for their time. In both phases, participants had access to information on relevant support services if required.

### Data analysis

Analysis was undertaken in three phases. First, we undertook a descriptive analysis of the trauma characteristics and other psychological variables in the sample. Using participant descriptions of their experiences, trauma types were categorized as interpersonal or non‐interpersonal and trauma exposure was categorized as either direct or indirect. For correlations, we used an alpha of .001 given the large number of variables to minimize type 1 errors. Next, correlation analyses investigated relationships between all key study variables, specifically, trauma characteristics (trauma type, exposure type, time since the most severe trauma), self‐reported mental health difficulties, MFS, DASS‐21, PCL‐5, PSS‐10, IES‐R, CRF, ERF and VAS scores. We then assessed whether VAS, CRF, ERF, PSS‐10 (day 7) and IES‐R (day seven) scores were associated with prior trauma characteristics, mental health difficulties and MFS scores with multiple regressions. Finally, hierarchical regression analysis was performed to assess whether VAS (baseline to post‐trial) and CRF scores were associated with subsequent VAS (post‐evidence), ERF, PSS‐10 (day seven) and IES‐R (day seven) scores, controlling for baseline trauma characteristics, self‐reported mental health difficulties, MFS, DASS‐21 and PCL‐5 scores, and baseline PSS‐10 and IES‐R scores. Separate analyses relating to the influence of evidence modality on stress and emotional responses, and verdict decisions, are reported elsewhere (Fawcett et al., in preparation).

## RESULTS

### Sample trauma characteristics

All participants in the sample reported trauma exposure, with a wide array of trauma exposures noted as their most stressful event, shown in Table 1. Bereavement, vehicle accidents and sexual assault were the most endorsed events perceived as severe among participants. The participants' most severe (traumatic) event occurred when participants were, on average, aged 27.17 years (SD = 13.20; range 6–73 years). For a third of participants (33.9%), the most severe event was interpersonal in nature and involved criminal victimization (30.6%). Three‐quarters (76.1%) of participants were directly exposed to the event. Almost half of the participants (46.1%) reported mental health difficulties, with most of these participants (86.7%) accessing support in relation to their symptoms.

**TABLE 1:** Most severe traumas reported by participants.

Trauma experience	*N* (%)
Involving death (family member/co‐worker/stranger)	47 (26.1%)
Motor incident/road traffic collision	21 (11.7%)
Involving sexual assault^a^	21 (11.7%)
Illness/injury of a loved one (non‐violent)	14 (7.8%)
Domestic abuse^a^	12 (6.7%)
Suicide/attempted suicide (of self or other)	12 (6.7%)
Physical/violent assault/threats of violence^a^	10 (5.6%)
Interpersonal difficulties (divorce, marriage breakdown, custody disputes)^a^	10 (5.6%)
Illness/injury of self (non‐violent)	10 (5.6%)
War/civil issues or conflict^a^	5 (2.8%)
Bullying^a^	3 (1.7%)
House fire	2 (1.1%)
Victim of armed robbery^a^	2 (1.1%)
Natural disaster	2 (1.1%)
Involving kidnapping^a^	2 (1.1%)
Negative accusations against self^a^	1 (.6%)
Terrorism^a^	1 (.6%)
Occupational difficulties (losing job, failing career)	1 (.6%)
Abortion	1 (.6%)
Crowd crushing	1 (.6%)
Family member perpetrating crime^a^	1 (.6%)
Fear of fireworks	1 (.6%)

^a^ Coded as interpersonal trauma.

### Prior trauma characteristics, medical fear, mental health and baseline measures

Descriptive statistics and correlations between key study variables are presented in Tables 2 and 3, respectively. Trauma type and exposure were unrelated to any baseline mental health variables at the *p* < .001 level. Time since the most severe trauma was significantly and negatively associated with baseline DASS‐21 and PSS‐10 scores at the bivariate level. Mental health difficulties were significantly and positively related to DASS‐21 and PSS‐10 scores at the bivariate level. MFS scores were positively associated with baseline PSS‐10 scores.

**TABLE 2:** Descriptives for key study variables.

Variable	*M*	SD	Min.	Max.
Time since trauma (months)	132.79	143.22	.05	720.00
MFS	15.21	9.30	.00	45.00
VAS (pre‐study)	19.11	21.52	.00	90.00
PCL‐5	15.87	15.63	.00	69.00
DASS‐21	11.10	11.06	.00	47.00
IES‐R (baseline)	12.21	15.86	.00	69.00
PSS‐10 (baseline)	16.85	6.95	1.00	33.00
VAS (pre‐trial)	21.93	21.44	.00	87.00
VAS (post‐trial)	20.95	20.08	.00	87.00
CRF	10.63	7.67	.00	37.00
VAS (post‐evidence)	25.08	22.13	.00	100.00
ERF	21.69	7.49	11.00	42.00
VAS (end of phase one)	24.92	23.44	.00	100.00
PSS‐10 (day seven)	21.01	5.33	10.00	32.00
IES‐R (day seven)	36.01	15.33	22.00	81.00

Abbreviations: CRF, Case Reactions Form; DASS‐21, Depression, Anxiety and Stress Scales; ERF, Evidence Reactions Form; IES‐R, Impact of Events Scale‐Revised; Max., maximum score; MFS, Medical Fear Survey; Min., minimum score; PCL‐5, PTSD Checklist for DSM‐5; PSS‐10, Perceived Stress Scales‐10; VAS, visual analogue scale for subjective stress.

**TABLE 3:** Correlations between key study variables.

	Trauma type^a^	Trauma exposure^a^	Time since most severe trauma	Medical fear	Mental health difficulties^a^
VAS (pre‐study)	−.04	.08	−.02	.07	.16
PCL‐5	−.13	.05	−.11	.17	.17
DASS‐21	−.12	.07	−.24***	.16	.26***
IES‐R (baseline)	−.05	.03	−.21	.22	.14
PSS‐10 (baseline)	−.06	.03	−.29***	.25***	.25***
VAS (pre‐trial)	−.05	.08	−.06	.12	.18
VAS (post‐trial)	.04	.00	−.13	.23	.16
CRF	.14	−.14	−.25***	.43***	−.07
VAS (post‐evidence)	.00	.02	−.14	.24***	.11
ERF	.10	−.17	−.26***	.39***	−.06
VAS (end of phase one)	.08	−.06	−.18	.27***	.06
PSS‐10 (day seven)	−.08	.14	.00	.07	.14
IES‐R (day seven)	−.11	.11	−.23	.17	.32***

Abbreviations: CRF, Case Reactions Form; DASS‐21, Depression, Anxiety and Stress Scales; ERF, Evidence Reactions Form; IES‐R, Impact of Events Scale‐Revised; PCL‐5, PTSD Checklist for DSM‐5; PSS‐10, Perceived Stress Scales‐10; VAS, visual analogue scale for subjective stress.

^a^ Trauma type, trauma exposure and mental health difficulties were dichotomised with higher values corresponding to interpersonal trauma, direct trauma and presence of prior mental health difficulties, respectively.

*** *p* < .001.

### Prior trauma characteristics, medical fear, mental health and immediate stress and emotional reactivity

Correlations (see Table 3) and regression analyses (shown in Table 4) were conducted to assess relationships between trauma characteristics, mental health and scores on the MFS, DASS‐21, PCL‐5, PSS‐10 and IES‐R, with immediate stress and emotional responses assessed using the VAS (post‐case and post‐evidence), CRF and ERF. Time since trauma was negatively related to CRF and ERF scores. MFS scores were positively related to CRF and ERF scores, and VAS post‐trial scores and at the end of phase one.

**TABLE 4:** Multiple regression analyses with immediate and follow‐up stress and emotional responses as the criterion.

	CRF (post‐case)	VAS (post‐case)	ERF (post‐evidence)	VAS (post‐evidence)	PSS‐10 (day seven)	IES‐R (day seven)
*F*	8.10***	5.73***	6.58***	3.39***	.99	18.35***
Adj. *R* ^2^	.26	.19	.26	.11	.00	.54
	β					
Trauma type^a^	−.10	−.12	.08	−.06	.03	.08
Trauma exposure^a^	−.08	−.01	−.16*	.02	.10	−.04
Time since trauma (months)	−.16*	−.01	−.21**	−.05	−.03	−.01
Prior mental health difficulties^a^	−.14	.06	−.11	.02	.11	.15
MFS	.37***	.17*	.36***	.19*	.10	.15*
DASS‐21	−.03	.22	−.13	.18	−.02	.16
PCL‐5	.13	.15	.15	.06	.04	.19
PSS‐10 (baseline)	.20*	.25**	.10	.18	.05	.01
IES‐R (baseline)	−.06	−.29*	.00	−.16	−.28	.42***

Abbreviations: CRF, Case Reactions Form; DASS‐21, Depression, Anxiety and Stress Scales; ERF, Evidence Reactions Form; IES‐R, Impact of Events Scale‐Revised; MFS, Medical Fear Survey; PCL‐5, PTSD Checklist for DSM‐5; PSS‐10, Perceived Stress Scales‐10; VAS, visual analogue scale for subjective stress.

^a^ Trauma type, trauma exposure and prior mental health difficulties were dichotomised with higher values corresponding to interpersonal trauma, direct trauma and presence of prior mental health difficulties, respectively.

* *p* < .05.

** *p* < .01.

*** *p* < .001.

Direct exposure to trauma (*p* = .025) and time since trauma (*p* = .004) were negative predictors of ERF scores. MFS scores were a significant positive predictor of VAS scores post‐trial (*p* = .018) and post‐evidence (*p* = .012), and CRF and ERF scores (both *p* < .001). Baseline PSS‐10 scores were positive predictors of CRF (*p =* .027) and VAS post‐case (*p* = .006) responses. IES‐R baseline score was a negative predictor of post‐trial stress (*p* = .013).

### Prior trauma characteristics, medical fear, mental health and stress responses at follow‐up

At baseline, 180 participants provided data, with 132 complete responses on day seven (73.3% completion rate). Preliminary analyses indicated that day seven participants did not differ on any demographic, trauma characteristic, MFS scores or mental health difficulties (all *p*s ≥ .115). Correlation analyses (see Table 3) revealed IES‐R scores on day seven were significantly and positively associated with reported mental health difficulties. There was a significant increase in IES‐R scores [*t*(131) = 22.28, *p* < .001, *d* = 1.94] between baseline and day seven, with the average score at day seven being three times that at baseline. Using suggested cut‐offs for probable PTSD diagnoses (Creamer et al., 2003), 10.6% of the sample at baseline had a total IES‐R score ≥33, which increased four‐fold to 44.7% at day seven. PSS‐10 scores also significantly increased from baseline to day seven [*t*(98) = 5.06, *p <* .001, *d* = .51].

Simultaneous multiple regression analyses (see Table 4) were conducted with trauma characteristics, mental health, MFS, DASS‐21 and baseline PSS‐10 and IES‐R scores as predictors of PSS‐10 and IES‐R scores at day seven. In the model with day seven IES‐R scores as the criterion reported mental health difficulties (*p* = .019) and baseline IES‐R scores (*p* < .001) were significant positive predictors. The second model with PSS‐10 scores as the criterion was non‐significant, explained no variance and had no significant predictors.

### Early stress responses on subsequent stress and emotional reactivity

Analysis of incremental changes in VAS scores at the five time points across phase one (with an adjusted alpha of *p* < .001 for multiple comparisons) revealed significant increases in VAS scores from prior to completing the baseline measures to pre‐trial [*t*(179) = 4.27, *p* < .001, *d* = .32], and also from post‐trial to the end of phase one [*t*(179) = 4.57, *p* < .001, *d* = .34]. Furthermore, VAS scores significantly increased from prior to completing the baseline measures to the end of phase one [*t*(179) = 3.76, *p* < .001, *d* = .28]. From the start to the end of phase one, VAS scores decreased for 22.7% participants, increased for 53.9% and returned to baseline for 23.3%. All VAS scores were significantly and positively related to one another (*p*s < .001). Participants endorsed a wide range of emotional responses on the measures, including anxious/distressed, uncomfortable and disgust.

Hierarchical multiple regressions were conducted to assess the influence of VAS and CRF scores on subsequent ERF and VAS (post‐evidence) scores, and PSS‐10 and IES‐R responses at day seven (see Table 5). Baseline trauma and mental health characteristics were entered in step one as control variables, with CRF scores and a VAS change score entered in step two, which was created by subtracting the first baseline VAS measurement from the VAS post‐trial score. In the first model with ERF scores as the criterion, trauma exposure (*p* = .025), time since trauma (*p* = .004) and MFS scores (*p* < .001) were significant predictors of ERF scores in step one. The inclusion of CRF (*p* < .001) and VAS change scores (*p* = .663) in step two resulted in an additional 39.7% of the variance in ERF scores being explained by the variables, totalling 63.3%. Trauma type (*p* = .002) and trauma exposure (*p* = .043) were significant predictors of ERF scores, and time since trauma and MFS were now non‐significant predictors. The second regression with VAS (post‐evidence) scores as a criterion explained 15% of the variance. Only VAS change from baseline to post‐trial was a significant predictor of VAS (post‐evidence) scores (*p* = .036). The third regression with PSS‐10 (day seven) scores as a criterion explained little variance (4.9%), with MFS scores in step one (*p* = .012) and VAS change scores from baseline to post‐trial in step two being significant predictor of later stress (*p* = .036). In the final regression with IES‐R (day seven) scores as criterion, prior mental health difficulties in step one (*p* = .019) and two (*p* = .013), and baseline IES‐R score (*p* < .001) at step two, were significantly related to IES‐R (day seven) scores.

**TABLE 5:** Results of hierarchical multiple regressions with emotional reactivity (post‐evidence) and stress responses at follow‐up.^a^

	ERF	VAS (post‐evidence)	PSS‐10 (day seven)	IES‐R (day seven)
Step 1				
Trauma type^b^	.08	−.06	.03	−.05
Trauma exposure^b^	−.16*	.02	.10	.08
Time since trauma (months)	−.21**	−.05	−.03	−.04
Prior mental health difficulties		.02	.11	.15*
MFS	.36***	.19**	.10	−.10
DASS‐21	−.13	.18	−.02	.16
PCL‐5	.15	.06	.04	.19
PSS‐10 (baseline)	.10	.18	.05	.07
IES‐R (baseline)	.00	−.16	−.28	.42***
Adj. *R* ^2^	.22	.11	.00	.54
*F*	6.58***	3.39***	.99	18.35***
Step 2				
Trauma type	.15**	−.04	.04	−.04
Trauma exposure	−.10*	.04	.12	.08
Time since trauma (months)	−.09	.00	.05	−.05
Prior mental health difficulties	−.01	.05	.15	.16*
MFS	.08	.10	−.01	−.04
DASS‐21	−.11	.18	.01	.14
PCL‐5	.06	.02	.02	.19
PSS‐10 (baseline)	−.06	.18	.06	−.02
IES‐R (baseline)	.05	−.16	−.34	.43***
VAS change (baseline to post‐trial)	−.02	.16*	.23*	−.08
CRF	.76***	.15	.15	.09
Adj. *R* ^2^	.63	.15	.05	.55
Δ*R* ^2^	.40	.05	.06	.01
Δ*F*	97.00***	4.85**	3.32*	1.18

Abbreviations: Adj. *R* ^2^, adjusted R‐square; CRF, Case Reactions Form; DASS‐21, Depression, Anxiety and Stress Scales; ERF, Evidence Reactions Form; IES‐R, Impact of Events Scale‐Revised; MFS, Medical Fear Survey; PCL‐5, PTSD Checklist for DSM‐5; PSS‐10, Perceived Stress Scales‐10; VAS, visual analogue scale for subjective stress; Δ*F* = *F* change; ΔR^2^ = *R*‐square change.

^a^ Standardized coefficients are reported for the predictors.

^b^ Trauma type, trauma exposure and prior mental health difficulties were dichotomised with higher values corresponding to interpersonal trauma, direct trauma and presence of prior mental health difficulties, respectively. ΔR^2^ = *R*‐square change; Δ*F* = *F* change.

* *p* < .05.

** *p* < .01.

*** *p* < .001.

## DISCUSSION

To our knowledge, this was the first study to examine the role of prior trauma and mental health characteristics on mock juror emotional and stress responses both during a trial and 1 week later, following exposure to distressing case material and presentation of skeletal evidence. Many participants reported interpersonal and direct trauma exposure as their most severe trauma and almost half self‐reported mental health difficulties. We found mixed support for our hypotheses. Traumatic stress symptoms in relation to participant's index trauma assessed using the IES‐R significantly increased over the study period, with the average PTSD symptom score at follow‐up being three times higher than that at baseline. Self‐reported mental health difficulties and medical fear were positively associated with some stress and emotional responses throughout the trial, although inconsistent findings were observed concerning trauma characteristics, stress and emotional reactivity. Finally, changes in VAS scores from baseline to post‐trial, and CRF scores, were found to relate to VAS (post‐evidence), PSS‐10 (day seven) and ERF scores over and above pre‐existing trauma characteristics and baseline measures of perceived stress and mental health (MFS, DASS‐21, PCL‐5, IES‐R).

### Pre‐existing stress symptoms and early stress responses can exacerbate trial stress

We found a significant increase in PTSD‐type symptoms on the IES‐R from baseline to 7 days later, with the average PTSD score being three times higher and 44% of the sample meeting PTSD‐type thresholds at day seven, compared with 11% at baseline. The follow‐up prevalence of PTSD symptoms is similar to that reported in a prior review of juror stress and well‐being, where up to 50% of jurors endorsed symptoms of traumatic stress (Lonergan et al., 2016). This suggests that the pre‐existing traumatic stress symptoms in relation to index trauma experienced by the mock jurors may be further exacerbated by exposure to distressing trial materials. Although the current study is advantageous in assessing symptoms post‐trial to identify sustained psychological aftereffects, it is important to note that the seven‐day follow‐up period was short, and we cannot tell if the symptoms naturally subsided or persisted. A longer timeframe could raise awareness of any chronic or enduring responses that may inform intervention efforts for jurors. For instance, some research has identified PTSD‐type symptoms in 16% of jurors (*N* = 62) up to 3 months post‐trial (Palmer, 2005). Furthermore, the IES‐R measures symptoms over the past 7 days, while the PCL‐5 measures symptoms over the past month, and thus at the follow‐up, there is a three‐week overlap in symptoms.

A further finding was that mock jurors who reported increases in VAS stress scores after being presented with case evidence from baseline were also more likely to report enhanced stress reactivity on the VAS (post‐evidence) and on the PSS‐10 administered 1 week later, regardless of prior trauma characteristics and responses on the baseline measures. These findings are consistent with a vulnerability‐stressor perspective (Gould et al., 2021; Ingram & Luxton, 2005), in that the stress of taking part in a trial can exacerbate pre‐existing traumatic stress symptoms in jurors in relation to their index trauma, but also early stress symptoms experienced during a trial can influence later emotional responses to subsequent traumatic material. Exposure to additional incidences of potentially traumatic material and graphic evidence may trigger reminders of historical personal experiences (Claunch, 2023), and exacerbate prior symptoms that are magnified throughout a trial. However, we do recognize that other social and environmental factors may contribute to juror distress, including sequestration (Robertson et al., 2009) and the courtroom environment itself (Carline et al., 2024), which future research should explore.

### Medical fear, mental health, stress and emotional reactivity

Mock jurors with self‐reported mental health difficulties endorsed more stress symptoms on the VAS pre‐ and post‐trial, and higher levels of PTSD symptoms in relation to their index trauma 7 days later. No extant research has explored pre‐trial mental health as a risk factor for stress symptoms in jurors, but given that mental health difficulties can be associated with challenges in managing emotional and stress responses (Berking & Wupperman, 2012), prior mental health characteristics may make some jurors more vulnerable to subsequent distress. Post‐trial, a minority of jurors can report moderate to clinically severe PTSD‐type symptoms, which may persist, deteriorate or recede after several weeks (Lonergan et al., 2016). More research is needed to understand whether pre‐existing mental health symptoms could be a risk factor for juror well‐being during trials.

The study highlighted that many potential jurors present with trauma histories and mental health needs, and has shown that these characteristics may influence stress and emotional responses during a trial. However, research on jury samples in England and Wales is challenging due to legal restrictions that prevent researchers from talking to jurors or examining court proceedings while trials are taking place. More research is needed to fully understand the psychological consequences associated with participation in the jury system, whether it be as a juror or other court professionals, to replicate efforts elsewhere internationally (e.g. Burton & Paton, 2021; James, 2020; O'Sullivan et al., 2022). Moreover, the potential impact of enhanced stress on juror decision making and verdict should be explored, given prior associations between stress and impaired cognitive decision making (Phillips‐Wren & Adya, 2020).

### Trauma characteristics, stress and emotional reactivity

Mixed findings were observed with respect to trauma characteristics, stress and emotional responses. At the bivariate level, trauma type and exposure were not related to any psychological responses. Time since trauma was negatively related to ERF scores at the bivariate and multivariate level, although the effects were weakened when controlling for CRF responses. Furthermore, interpersonal trauma and those with indirect trauma exposure displayed more intense emotional reactions to the skeletal evidence when accounting for medical fear and other mental health characteristics. These findings contrast with previous work that finds no relationship between trauma characteristics and stress responses among jurors (Palmer, 2005), and is inconsistent with qualitative research which suggested that prior trauma exposure overall may be a risk factor for juror well‐being (Antonio, 2008). The mixed findings may be an artefact of a recall bias, or difficulties measuring time since a traumatic event as different coding methods are used in the wider trauma literature (Szabo et al., 2017). However, as subjective characteristics of trauma (e.g. controllability, valence) may better relate to psychological outcomes compared to objective characteristics (Luhmann et al., 2021), the findings call for the investigation of third variables that may explain relationships with emotional responses to distressing evidence among jurors. Our findings also point to the need for more qualitative work to unpick the nuances of prior trauma exposure on juror well‐being to better inform support efforts.

### Practical implications

Given the increase in stress symptoms over the course of the study, particularly the significant increase in PTSD symptoms from baseline to follow‐up, there is a clear need to facilitate access to immediate post‐trial support for jurors experiencing psychological distress. Currently, in England and Wales, no formal pre‐trial preparation is available. During a trial, jurors are told not to discuss their experiences with family members, friends, employers or mental health professionals. Even after a trial has finished, jurors are never permitted to discuss courtroom deliberations. Not permitting discussions about the trial may limit jurors' imaginal exposure to traumatic elements of it, thus blocking them from processes which could lead to reduction in trauma‐related distress (Zoellner et al., 2023).

Jurors, including those with prior trauma history, may not be fully prepared for potential emotional distress that a trial may bring (Claunch, 2023). Jurors may benefit from strategies instilling psychological preparedness, as the latter can buffer the effects of direct (Başoğlu et al., 1997) and indirect (Livanou et al., 2023) exposure to trauma. Psychological preparedness involves having prior knowledge, training and readiness for trauma exposure, a ‘mind‐set’ reflecting commitment to a cause and less endorsement of ‘basic assumptions’ about the world being a safe and just place (Janoff‐Bulman, 1992; Livanou et al., 2023). Future studies need to examine the extent to which jurors' basic assumptions are associated with their emotional responses to cases and skeletal evidence. This support is similar to initiatives already provided to professionals working in hostile environments (Nowlan, 2014), however, research is required as to what information should be provided and how this information should be delivered within a courtroom context. It may involve educating jurors about various stress responses and how these may manifest, as well as providing ways to help manage any symptoms experienced.

Findings from this study could also help to identify some jurors who may be at increased risk of stress both during and post‐trial, which may be due to prior mental health and trauma experiences. Previous research that has explored the potential for dedicated post‐trial support has shown that jurors would value specialist input, rather than more generic provision (Thomas, 2020), which is being increasingly reflected in practice. Scotland and the Australian state of Victoria have dedicated free counselling services for jurors (Victoria, 2024; BPS, 2023), and Canada offers peer support programmes (Canadian Juries Commission, 2023). In England and Wales, a pilot for well‐being support post‐trial is underway (Ministry of Justice et al., 2024). Getting professional help to overcome the emotional impact of being a juror might be limited by the fact that they are not allowed to discuss potentially traumatic details relating to the trial. However, eye‐movement desensitization and reprocessing therapy (EMDR) could be helpful in this context, as apart from being evidence‐based, it does not necessarily require full disclosure of the trauma (Wilson et al., 2018).

Alongside support for jurors, we echo previous calls (Ellison & Munro, 2017) to provide training to court staff, including judges, barristers and court clerks, around mental health and vicarious trauma. This would help court professionals to recognize stress symptoms in jurors, but also raise awareness of the impact of vicarious trauma upon themselves working in courtroom settings. Once the evidence base in this field grows further, the development of best practice guidance could also help to work towards a more trauma‐informed courtroom which responds appropriately to jurors, and others, who have experienced trauma directly or indirectly.

## LIMITATIONS

This research has several limitations. The research featured a mock juror scenario in which most participants did not have prior experience of jury duty, so the results may not reflect the views of jurors during a live trial (Thomas, 2020), although the results are broadly in line with similar studies that have recruited previous jurors through community surveys (Robertson et al., 2009; Welsh et al., 2020). Participants were exposed to traumatogenic materials and reached verdicts individually, which did not reflect real‐life jury scenarios. This was based on limited study resources and the study focus being to examine the presentation of evidence on juror well‐being. In a real‐life courtroom, jurors are tasked to reach a verdict through deliberation with other jurors. Vulnerability‐stressor perspectives (Ingram & Luxton, 2005) emphasize interactions between individual predisposing factors and the relational context. However, legal restrictions preventing jurors from discussing deliberations mean that researchers are unable to collect data to explore these social influences on juror stress and emotional responses. It is possible the prevalence of PTSD and trauma were overestimated, although they are congruent with data from jurors highlighted in a systematic review of this area (Lonergan et al., 2016). The nature of the research design required that the entire sample had prior trauma exposure. It is possible that those who were interested in the research or perceived themselves to be emotionally resilient are over‐represented in the study, which is not representative of all trauma survivors or people who do not report prior trauma exposure.

## CONCLUSION

Jury duty can be a positive and rewarding experience for most jurors, despite potential exposure to disturbing evidence and information during a trial. We found that some prior self‐reported trauma and mental health characteristics, and early stress reactions, may be related to stress and emotional responses at various stages of a trial, which could further compound the stress from prior traumatic experiences and jury service. Acknowledging and addressing the support needs of jurors could enhance their well‐being and enable them to fulfil this important civic duty.

## AUTHOR CONTRIBUTIONS

**Matthew Brooks:** Formal analysis; writing – original draft; writing – review and editing. **Jessica Glynn:** Investigation; writing – original draft; writing – review and editing. **Hannah Fawcett:** Conceptualization; methodology; project administration; writing – review and editing; investigation; funding acquisition. **Aminah Barnes:** Investigation. **Rachael Carew:** Resources. **David Errickson:** Resources. **Maria Livanou:** Conceptualization; methodology; resources; writing – review and editing.

## CONFLICT OF INTEREST STATEMENT

The authors have no known conflicts of interest.

## Data Availability

The data and some materials from this study are available on the Open Science Framework: https://osf.io/x4k8m/.
